# SMAD4–BPIFA1 Axis Governs Airway Antiviral Defense in Myhre Syndrome

**DOI:** 10.3390/biom16081118

**Published:** 2026-07-31

**Authors:** Yuanpu P. Di, Bodong Wen, Fengyuan Li, Felicia Tang, Dena Shen, Mark E. Lindsay, Angela E. Lin, Yin Chen, Hongmei Mou

**Affiliations:** 1 Department of Cellular and Molecular Medicine, Center for Translational Science, Florida International University, Port St. Lucie, FL 34987, USA; ydi@fiu.edu; 2 Mucosal Immunology and Biology Research Center, Massachusetts General Hospital, Boston, MA 02114, USA; bwen6@jhu.edu (B.W.); charlesli.1037@gmail.com (F.L.); fel81012@gmail.com (F.T.); denashen2028@cdnis.edu.hk (D.S.); 3 Cardiovascular Genetics Program, Massachusetts General Hospital, Boston, MA 02114, USA; lindsay.mark@mgh.harvard.edu; 4 Cardiovascular Research Center, Department of Medicine, Massachusetts General Hospital, Boston, MA 02114, USA; 5 Medical Genetics, Department of Pediatrics, Mass General for Children, Boston, MA 02114, USA; alin@mgb.org; 6 Department of Pharmacology and Toxicology, School of Pharmacy, The University of Arizona, Tucson, AZ 85721, USA; ychen@pharmacy.arizona.edu

**Keywords:** Myhre syndrome, SMAD4, TGF-β signaling, gain-of-function, rare disease, BPIFA1, BPIFA1-derived peptides, respiratory syncytial virus, innate immunity, airway epithelium

## Abstract

Myhre syndrome is a rare autosomal-dominant disorder caused by gain-of-function (GOF) variants in SMAD4. Although it is primarily recognized as a fibrotic disease, we and others have identified respiratory infection-associated morbidity as a major clinical burden. Regardless, the epithelial-intrinsic mechanisms underlying increased viral susceptibility in Myhre syndrome remain poorly understood. Here, using patient-derived nasal airway epithelial cells (NECs) and respiratory syncytial virus (RSV) infection as a model, we demonstrate that infection is markedly exacerbated in Myhre syndrome epithelium relative to matched healthy controls. We further show that activation of TGF-β1–SMAD4 signaling in healthy donor NECs phenocopies augmented RSV susceptibility, identifying SMAD4 signaling as a bona fide pro-viral pathway in airway epithelium. Mechanistically, we identify a significant reduction in BPIFA1 expression in Myhre syndrome NECs. BPIFA1 is a key secreted innate defense protein with antimicrobial, immunomodulatory, and emerging antiviral functions. SMAD signaling suppresses BPIFA1 expression, and its knockdown in healthy cells mimics the increased viral susceptibility, linking SMAD4 gain-of-function to impaired epithelial immunity. Therapeutically, we evaluated novel, stabilized 24-amino acid BPIFA1-derived peptides as a potential intervention. Treatment with active peptides (A4-153 and A4-X7) significantly reduced RSV infectivity and epithelial damage in both healthy and Myhre syndrome airway epithelial cultures, whereas a scrambled control peptide (A4-198) showed no significant effect. Collectively, our findings establish that GOF SMAD4 signaling enhances viral vulnerability in airway epithelium through suppression of BPIFA1-mediated antiviral defense and demonstrate that BPIFA1-derived peptide therapeutics represent a promising strategy to restore innate antiviral protection.

## 1. Introduction

Myhre syndrome (first described by Dr. Myhre in 1981) is an ultra-rare disorder caused by presumed gain-of-function (GOF) mutations in the SMAD4 gene [1,2,3,4]. SMAD4 functions as a central mediator of TGF-β superfamily signaling by binding activated receptor SMADs in the cytoplasm and facilitating their translocation to the nucleus, where they regulate transcription [5]. Myhre syndrome is characterized by multisystem connective tissue involvement affecting the integumentary, cardiovascular, respiratory, gastrointestinal, and musculoskeletal systems [3,4,6,7]. Progressive fibroproliferative changes can occur spontaneously or follow invasive procedures [3,4,6,7]. Although Myhre syndrome is regarded as a fibrotic disease, over 7 years of follow-up in 47 patients, we reported that respiratory morbidity emerged as a defining and disproportionate clinical burden [6]. Recurrent upper respiratory tract infections, including otitis media, sinusitis, and mastoiditis, are among the most frequent and debilitating complications, contributing to progressive hearing loss, increased hospitalization, exacerbation of laryngotracheal stenosis and fibrosis, and reduced quality of life [6,8]. Despite these clinical observations, the epithelial-intrinsic mechanisms driving increased viral susceptibility in Myhre syndrome remain poorly understood.

The airway epithelium functions as the primary physical and immunological barrier between the host and inhaled particles or pathogens. Beyond its barrier function, the airway epithelium actively participates in innate immune surveillance through the secretion of antimicrobial proteins, mucins, and cytokines into the airway surface liquid (ASL). Disruption of this epithelial defense layer, whether through genetic, environmental, or pathogen-driven mechanisms, can markedly increase viral and bacterial susceptibility. Respiratory syncytial virus (RSV) is one of the most clinically significant respiratory pathogens, particularly in young children, immunocompromised individuals, and patients with underlying pulmonary disease [9,10], making it an ideal model pathogen to investigate the mechanistic basis of the heightened vulnerability to mucosal infections in Myhre syndrome.

TGF-β signaling has been previously implicated as a pro-viral pathway in multiple viral infections in respiratory systems [11,12,13,14,15,16,17,18]. In these studies, TGF-β has been shown to suppress interferon signaling, thereby weakening antiviral defenses; promote cytoskeletal remodeling that facilitates viral trafficking; and inhibit cellular proliferation while redirecting host machinery toward viral replication, collectively creating a permissive environment for infection [11,12,13,14,15,19,20]. However, it remains unclear whether additional epithelial-derived antiviral mediators are regulated by TGF-β signaling.

BPIFA1 (BPI fold-containing family A member 1; also known as SPLUNC1) is a secreted innate defense protein predominantly expressed by airway epithelial cells and serous cells of submucosal glands [21,22,23]. As a major component of the airway surface liquid (ASL), BPIFA1 performs multiple host defense functions, including antimicrobial and immunomodulatory activities, regulation of airway surface tension, and modulation of ion transport [22,23,24,25,26,27]. Emerging evidence also supports antiviral roles for BPIFA1 [23,28,29,30,31,32,33], although the underlying mechanisms remain incompletely defined. In the context of influenza A virus (IAV) infection, BPIFA1 has been shown to directly restrict viral binding to epithelial cells and to impede nuclear import of viral ribonucleoprotein complexes during early stages of infection [28]. In addition, BPIFA1 modulates innate immune signaling pathways, including interferon production and interferon-stimulated gene (ISG) responses, and influences immune cell recruitment—particularly neutrophil trafficking—through regulation of chemokine axes such as CXCL10 and IFN-γ [25]. Beyond immune regulation, BPIFA1 contributes to mucociliary clearance and maintenance of epithelial barrier integrity, further limiting viral propagation. We have previously reported that BPIFA1 expression is markedly reduced in nasal airway epithelial cells derived from patients with Myhre syndrome, accompanied by impaired innate immune responses to bacterial challenge [8]. Whether this BPIFA1 deficiency contributes to viral susceptibility, and whether it is mechanistically driven by aberrant SMAD4 signaling, has not been investigated.

In the present study, we use patient-derived nasal ALI cultures to systematically investigate the epithelial-intrinsic basis for heightened RSV viral susceptibility in Myhre syndrome. We demonstrate that GOF SMAD4 signaling suppresses BPIFA1 expression, and that BPIFA1 deficiency itself is sufficient to recapitulate the viral susceptibility phenotype observed in Myhre syndrome. Critically, we show that stabilized BPIFA1-derived peptides can restore antiviral defense in Myhre syndrome airway epithelial cultures, establishing a proof of concept for a novel peptide-based therapeutic strategy. Together, these findings illuminate a previously unrecognized SMAD4–BPIFA1 axis that governs antiviral epithelial immunity and opens new avenues for therapeutic intervention in Myhre syndrome and potentially in broader respiratory viral diseases.

## 2. Materials and Methods

### 2.1. Human Nasal Basal Cell Isolation, Expansion, and Air–Liquid Interface Differentiation

The nasal epithelial basal cells used in this study were de-identified and have been previously described [8]. The use of these cells was conducted in accordance with the Institutional Review Board (IRB)-approved protocol #2017P001479 at Massachusetts General Hospital, approved on March 16, 2026. The nasal epithelial cells were cultured in Small Airway Epithelial Cell Medium (Promocell, GmbH C21160, available through Fisher Scientific, St. Louis, MO, USA) supplemented with ROCK inhibitor (5–10 µM, Tocris Bioscience, Cat. No. 1254, Minneapolis, MN, USA), A-83-01 (1 µM, Tocris Bioscience, Cat. No. 2939, Minneapolis, MN, USA), and CHIR99021 (0.5 µM, Tocris Bioscience, Cat. No. 4423, Minneapolis, MN, USA) on laminin-enriched 804G-coated plates [34]. For differentiation, basal cells were seeded onto 0.4 µm 12-well transwell inserts (>6000 cells/mm^2^, Corning, Cat. No. 3460, Tewksbury, MA USA) to achieve full confluence, then cultured in PneumaCult-ALI medium (STEMCELL Technologies, Cat. No. 05001, Cambridge, MA, USA) [34]. After attachment, ALI conditions were established by removing apical medium. Medium was changed every two days, and differentiation was maintained for 16–21 days, with ciliation monitored by microscopy.

### 2.2. ALI Culture Immunofluorescence and Quantification

After mucociliary differentiation, transwell membranes were fixed in 4% paraformaldehyde (Thermo Fisher Scientific, Cat. No. J61899-AK, Waltham, MA, USA, 5–10 min, room temperature), washed, and permeabilized with PBS (Thermo Fisher Scientific, Cat. No. 10010023, Waltham, MA, USA) containing 0.2% Triton X-100 (Thermo Fisher Scientific, Cat. No. A16046-AE, Waltham, MA, USA. Membranes were subjected to whole-mount staining. Primary antibodies included BPIFA1 [21] (rabbit, lab-made, 1:1000), acetylated tubulin (mouse, 1:1000, T6793, Sigma-Aldrich, Burlington, MA, USA) for ciliated cells, MUC5AC (mouse, 1:100, MA5-12178, Thermo Fisher Scientific, Waltham, MA, USA) for goblet cells, cleaved caspase-3 (rabbit, 1:100, #9661, Cell Signaling Technology, Danvers, MA, USA), and ZO-1 (rabbit, 1:100, ab216880, Abcam, Waltham, MA, USA). Nuclei were stained with Hoechst (1:500, H3570, Thermo Fisher Scientific, Waltham, MA, USA). For secondary labeling, antibodies conjugated with Alexa Fluor (488, Cat. No. A-21202 and A-21206 and 594, Cat. No. A-21203, A-21207) were obtained from Life Technologies (Waltham, MA, USA) and used at a dilution of 1:500. Images were acquired using Nikon A1 confocal and ECLIPSE Ti2 microscopes and analyzed with NIS-Elements.

### 2.3. RSV Preparation and Infection of ALI Cultures

The RSV A2-GFP strain (ViraTree, Cat. No. R121, Durham, NC, USA) was propagated in HEp-2 cells (ATCC CCL-23, Manassas, VA, USA) as described previously [35]. Briefly, HEp-2 cells were cultured in DMEM/F12 medium (Thermo Fisher Scientific, Cat. No. A4192001, Waltham, MA, USA) supplemented with 2% FBS (Thermo Fisher Scientific, Cat. No. A5670201, Waltham, MA, USA) and 1% GlutaMAX (Thermo Fisher Scientific, Cat. No. 35050061, Waltham, MA, USA). At ~80% confluence, cells were infected with RSV (MOI 1.0) for 1 h at 37 °C. The cells were harvested at 4–5 dpi when cytopathic effects were evident. The cell debris was removed by centrifugation, and supernatants were aliquoted and stored at −80 °C. Viral titers were determined by plaque assay [36]. RSV infection experiments were performed in the biosafety level 2 (BSL-2) facility. In brief, RSV stocks were thawed and diluted in DMEM/F12 immediately before use. Before infection, ALI cultures were gently washed twice apically with PBS to remove mucus, then infection was performed with RSV (5–10 × 10^5^ PFU or MOI 1.0 in 150 μL per well in a 12-well Transwell plate) for 1 h at 37 °C. After infection, cultures were washed gently three times with PBS to remove unbound virus and maintained for downstream analysis. At endpoint, cultures were fixed in 4% paraformaldehyde for immunostaining.

### 2.4. BPIFA1 Treatment During RSV Infection

BPIFA1 peptides (A4-198, A4-153, and A4-X7) at the various concentrations were pre-incubated with RSV (5–10 × 10^5^ PFU or MOI 1.0 in 150 μL per well in a 12-well Transwell plate) for 20–30 min before infection. The peptide–virus mixtures were then added to the apical surface of ALI cultures and incubated for 1 h at 37 °C. Following infection, cultures were washed three times with PBS to remove unbound virus and maintained for downstream analyses at the indicated time points.

### 2.5. Statistics

All experiments were performed at least three times. For quantification, 4–8 random non-overlapping fields per sample were imaged (10× or 20×). Marker-positive cells were quantified and normalized to either the total number of nuclei or the total imaging area. Results are presented as the percentage of epithelial cells or imaging areas relative to control samples. Comparisons between two groups were analyzed using an unpaired Student’s *t* test. For multiple groups, one-way ANOVA with Tukey’s post hoc test or two-way ANOVA with Dunn’s multiple comparisons test was used as appropriate. Analyses were conducted using GraphPad Prism 11, and *p* < 0.05 was considered statistically significant.

## 3. Results

### 3.1. Myhre Syndrome Respiratory Epithelial Cells Exhibit Heightened Susceptibility to RSV Infection

**Figure 1:**
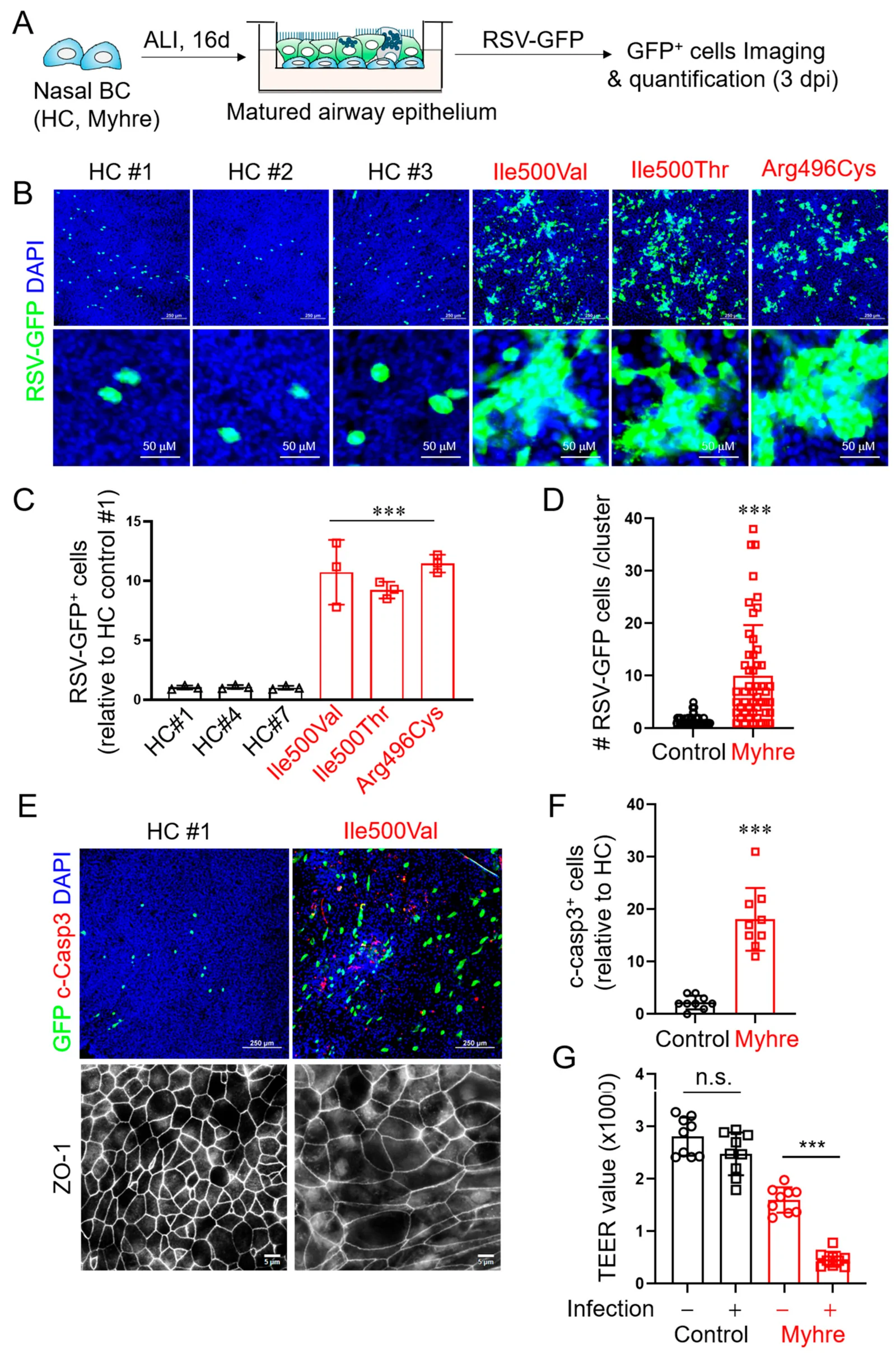
Myhre syndrome nasal respiratory epithelial cells exhibit heightened susceptibility to RSV infection. (**A**) Schematic illustration of the experimental design used to compare RSV infection in NECs derived from Myhre syndrome patients and healthy controls. (**B**) Fluorescence images showing increased RSV-GFP infection in Myhre syndrome NECs harboring SMAD4 variants (Ile500Val, Ile500Thr, Arg496Cys) compared to healthy controls. Scale bar: 250 μm. Higher-magnification images (bottom) reveal larger and more extensive RSV-GFP-positive clusters in Myhre syndrome cultures compared with healthy controls. Scale bar: 50 μm. (**C**) Quantification of RSV-GFP-positive cells per imaging field, normalized to healthy controls. Data are presented as mean ± SEM; *** *p* < 0.001; *n* = 3 independent experiments. (**D**) Quantification of RSV-GFP-positive cluster size, demonstrating increased viral spread in Myhre syndrome NECs. (**E**) Immunofluorescence staining of RSV-GFP with cleaved caspase-3 (apoptosis marker, top) and ZO-1 (tight junction marker, bottom) in healthy control #1 and Myhre syndrome (SMAD4-Ile500Val) NECs. Scale bars: 250 μm (top) and 5 μm (bottom). (**F**) Quantification of c-casp3–positive cells per imaging field, normalized to healthy controls, showing increased epithelial injuries in Myhre syndrome NECs. Data are presented as mean ± SEM; *** *p* < 0.001; *n* = 9 independent experiments. (**G**) Transepithelial electrical resistance (TEER) measurements in healthy control and Myhre syndrome NECs before infection and at 3 dpi with RSV, indicating impaired barrier integrity in Myhre syndrome NEC cultures. Data are shown as mean ± SEM; *** *p* < 0.001; n.s. Not significant. *n* = 9 independent experiments.

We recently reported that patients with Myhre syndrome exhibit an increased susceptibility to opportunistic respiratory infections, including recurrent otitis media, rhinosinusitis, and pneumonia [6,8]. However, the epithelial-mediated mechanisms underlying this vulnerability remain poorly defined. To address this, we employed RSV infection as a quantitative model to compare viral infectivity between nasal epithelial cells (NECs) derived from healthy individuals and patients with Myhre syndrome (Ile500Val, Ile500Thr, Arg496Cys) [8]. Age- and sex-matched NECs maintained at the air–liquid interface (ALI) were infected with RSV expressing green fluorescent protein (RSV-GFP) at a multiplicity of infection (MOI) of 1.0, and cultures were analyzed at 3 days post-infection (dpi) (Figure 1A).

Quantification of GFP-positive cells revealed that RSV exhibits markedly increased capacity to infect NECs derived from patients with Myhre syndrome relative to healthy controls, as evidenced by a significantly higher number of GFP^+^ cells (Figure 1B,C). Moreover, viral spread was enhanced in Myhre syndrome cultures, as indicated by the increased size and density of GFP^+^ cell clusters, consistent with more rapid cell-to-cell propagation of RSV (Figure 1B,D). RSV-infected cultures from Myhre syndrome patients also exhibited significantly greater epithelial injury at 3 dpi compared to healthy controls, as evidenced by an increased number of cleaved caspase-3-positive cells (Figure 1E,F), disrupted junctional localization of zonula occludens-1 (ZO-1) (Figure 1E), and reduced transepithelial electrical resistance (TEER) (Figure 1G). These findings establish that Myhre syndrome airway epithelium is intrinsically more susceptible to RSV infection and associated epithelial injury.

### 3.2. Activation of TGF-β/SMAD Signaling Augments RSV Infection Susceptibility in Airway Epithelial Cells

**Figure 2:**
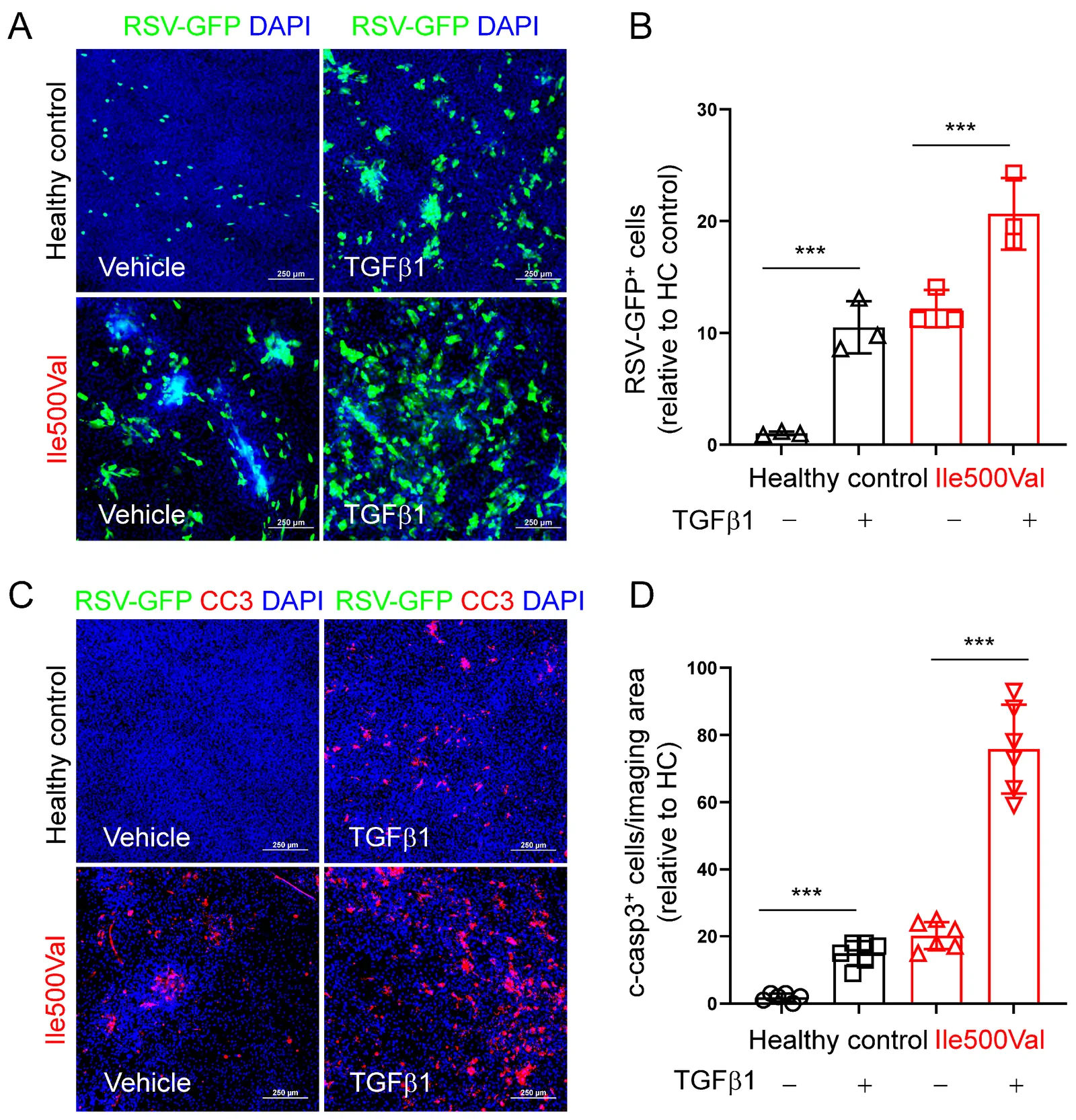
Activation of TGF-β/SMAD signaling enhances susceptibility to RSV infection in airway epithelial cells. (**A**) Immunofluorescence images showing RSV-GFP-infected NECs derived from a healthy control and a Myhre syndrome patient (SMAD4-Ile500Val) following treatment with vehicle or 10 ng/mL TGF-β. Scale bars, 250 μm. (**B**) Quantification of RSV-GFP-infected in NECs from a healthy subject and a Myhre syndrome patient (SMAD4-Ile500Val) with or without TGF-β treatment. Data are presented as mean ± SEM; *** *p* < 0.001; *n* = 3 independent experiments. (**C**) Immunofluorescence showing cleaved caspase-3-positive apoptotic cells in NECs derived from a healthy control and a Myhre syndrome patient (SMAD4-Ile500Val) following treatment with vehicle or 10 ng/mL TGF-β. Scale bars, 250 μm. (**D**) Quantification of cleaved caspase-3-positive cells per image in NECs from a healthy subject and a Myhre syndrome patient (SMAD4-Ile500Val) with or without TGF-β treatment. Data are presented as mean ± SEM; *** *p* < 0.001; *n* = 6 independent experiments.

Cellular and molecular analyses of NECs from patients with Myhre syndrome have demonstrated that GOF variants in SMAD4 enhance ligand-induced SMAD signaling and downstream transcriptional activity [8]. Based on this, we hypothesized that the heightened susceptibility to RSV infection in Myhre syndrome NECs is driven, at least in part, by hyperactivation of the TGF-β/SMAD signaling pathway. To test this hypothesis, NECs derived from healthy controls and patients with Myhre syndrome were treated with recombinant TGF-β1 (10 ng/mL) beginning 2 h prior to RSV-GFP infection and maintained throughout the course of infection. Cultures were analyzed at 3 dpi. The data showed that activation of TGF-β signaling significantly augmented RSV infectivity and enhanced viral spread in both healthy and Myhre syndrome NECs (Figure 2A,B). Furthermore, TGF-β1 treatment exacerbated epithelial injury, as reflected by elevated levels of cleaved caspase-3 (Figure 2C,D). These data demonstrated augmented SMAD signaling as a pro-viral mechanism that contributes to heightened RSV pathogenesis and establishes a mechanistic link between GOF SMAD4 signaling and increased viral susceptibility in Myhre syndrome.

### 3.3. Reduced BPIFA1 Expression Contributes to Increased Viral Susceptibility in Airway Epithelial Cells

**Figure 3:**
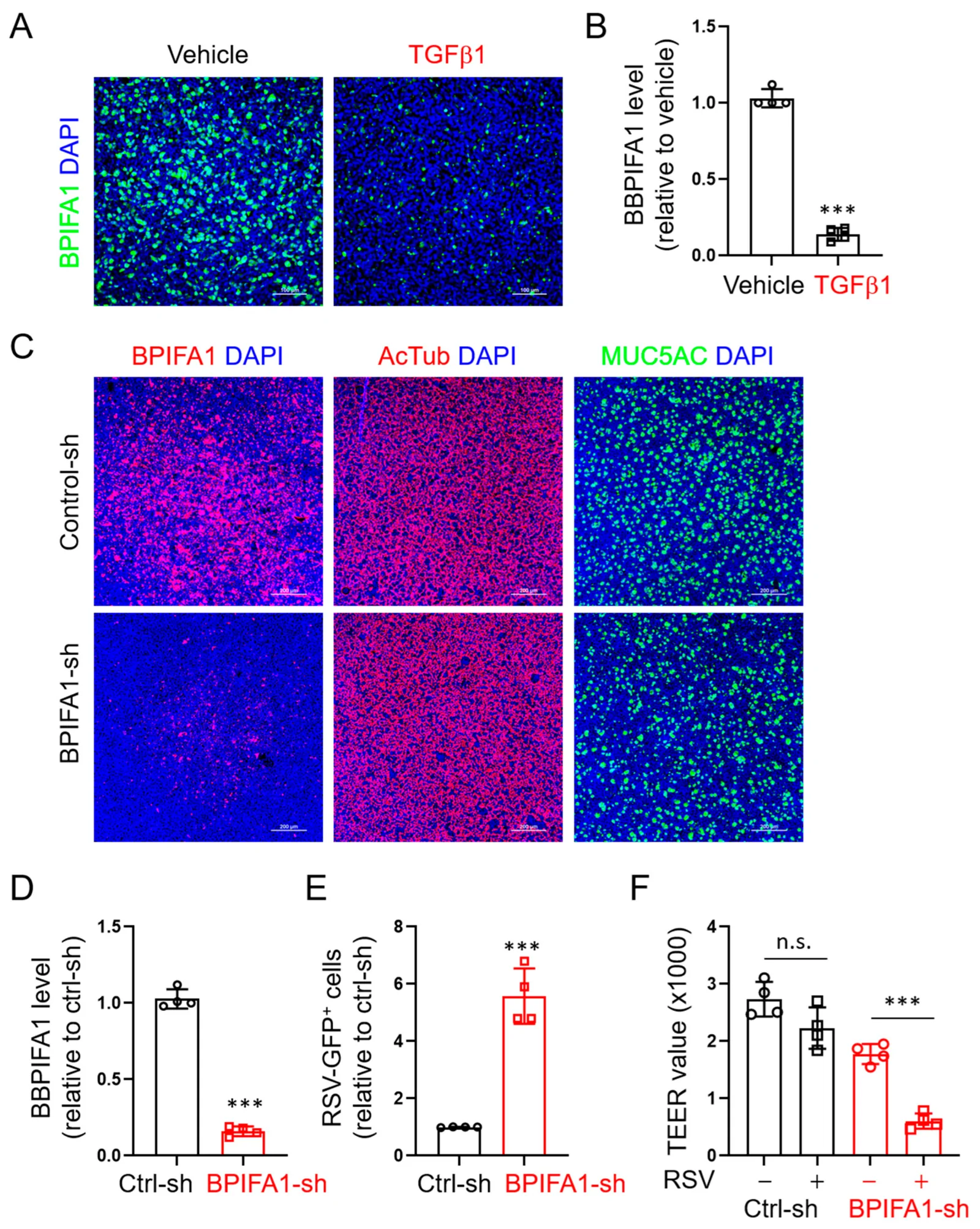
Reduced BPIFA1 expression contributes to increased viral susceptibility in airway epithelial cells. (**A**) Immunofluorescence images of BPIFA1 in healthy NECs demonstrate that TGF-β treatment significantly reduces BPIFA1 expression. Scale bars, 100 μm. (**B**) Quantification of BPIFA1 expression levels in healthy NECs treated with or without TGF-β. Data are presented as mean ± SEM; *** *p* < 0.001; *n* = 4 independent experiments. (**C**) Immunofluorescence images showing that shRNA-mediated BPIFA1 depletion in healthy NECs does not affect the differentiation of major airway epithelial cell types, including AcTub+ ciliated cells and MUC5AC+ goblet cells. Scale bars, 200 μm. (**D**) Quantification of BPIFA1 expression levels in healthy NECs treated with control shRNA and BPIFA1 shRNA. Data are presented as mean ± SEM; *** *p* < 0.001; n = 4 independent experiments. (**E**) Quantification of RSV-GFP-positive cells at 3dpi demonstrating enhanced RSV infectivity in NECs expressing BPIFA1 shRNA compared with control shRNA-expressing cells. Data are presented as mean ± SEM; *** *p* < 0.001; *n* = 4 independent experiments. (**F**) TEER measurements in NECs expressing BPIFA1 shRNA or control shRNA before infection and at 3 dpi with RSV, demonstrating impaired epithelial barrier integrity in BPIFA1-deficient NEC cultures. Data are presented as mean ± SEM; *** *p* < 0.001; n.s.: not significant. *n* = 4 independent experiments.

BPIFA1 is a secreted innate defense protein abundantly expressed by airway epithelial cells and enriched within the ASL at the host–pathogen interface. Beyond its established roles in antimicrobial defense, emerging evidence has attributed antiviral activity to BPIFA1. We have reported that BPIFA1 expression is significantly reduced in NECs from patients with Myhre syndrome and that this reduction is associated with impaired innate immune responses to bacterial infection [8]. To determine whether BPIFA1 suppression is mechanistically driven by aberrant SMAD signaling, we treated healthy NECs with TGF-β1. TGF-β1 activation resulted in significant suppression of BPIFA1 expression (Figure 3A,B). To directly assess the functional contribution of BPIFA1 deficiency to antiviral susceptibility, we performed shRNA-mediated knockdown of BPIFA1 in healthy NECs (Figure 3C,D). BPIFA1 deletion does not affect the differentiation of other major airway epithelial cell types, including AcTub+ ciliated cells, which are primary targets of RSV infection, and MUC5AC+ goblet cells (Figure 3C). However, RSV exhibited significantly increased infectivity in BPIFA1-depleted NECs and greater epithelial injury compared to scrambled shRNA controls (Figure 3E,F), demonstrating that BPIFA1 is a critical determinant of antiviral defense in airway epithelium.

**Figure 4:**
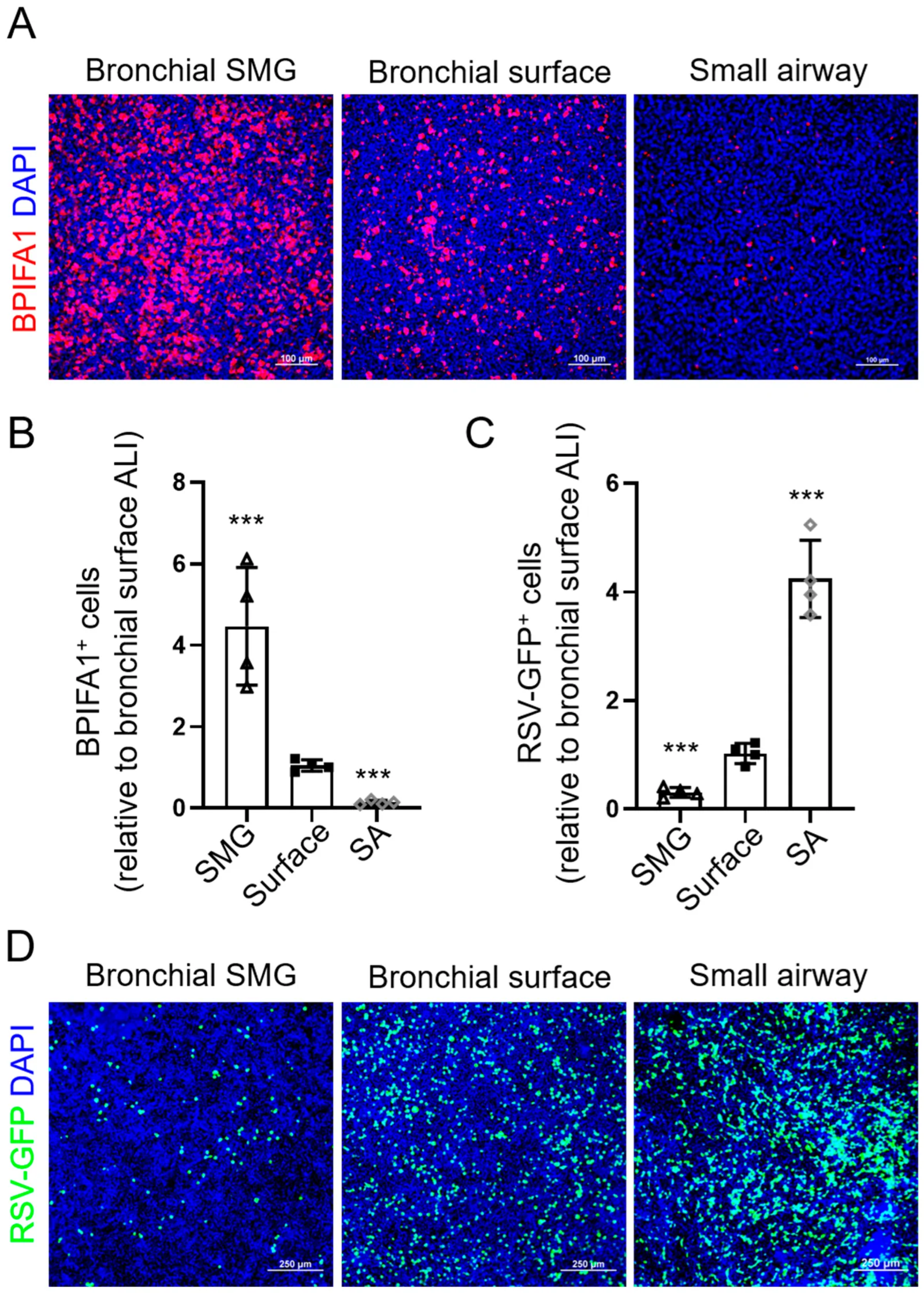
Spatial gradient of BPIFA1 expression along the human airways and its inverse relationship with viral susceptibility. (**A**) Airway basal cells isolated from bronchial SMGs, bronchial surface epithelium, and small airways were differentiated under ALI conditions. Immunostaining for BPIFA1 showed high expression in bronchial SMG ALI cultures, intermediate expression in bronchial surface ALI cultures, and minimal expression in small airway ALI cultures, consistent with their in vivo expression patterns. Scale bars, 100 μm. (**B**) Quantification of BPIFA1-positive cells in SMG, bronchial surface, and small airway ALI cultures, normalized to bronchial surface ALI cultures. Data are presented as mean ± SEM; *** *p* < 0.001; *n* = 4 independent experiments. (**C**) Quantification of RSV-GFP-positive cells demonstrating increased RSV infectivity in small airway ALI cultures, compared with bronchial surface and SMG ALI cultures. Data are presented as mean ± SEM; *** *p* < 0.001; *n* = 4 independent experiments. (**D**) Representative immunofluorescence images showing RSV-GFP-infected cells in SMG, bronchial surface, and small airway ALI cultures. Reduced numbers of RSV-GFP-positive cells were observed in SMG and bronchial surface ALI cultures compared with small airway ALI cultures. Scale bars, 250 μm.

We recently reported that BPIFA1 expression exhibits a distinct spatial gradient along the human airways, with the highest expression in the upper airway (nasopharyngeal, tracheal, and segmental bronchial regions) that progressively declines toward distal airway regions [21]. Furthermore, SMG epithelial cells express substantially higher levels of BPIFA1 than surface airway epithelial cells [21]. This regional distribution is faithfully recapitulated in our ALI cultures: bronchial SMG epithelial cultures exhibit the highest BPIFA1 expression, bronchial surface epithelial cultures display intermediate levels, and small airway epithelial cultures show minimal expression (Figure 4A,B). Consistent with an inverse relationship between BPIFA1 expression and viral susceptibility, RSV infectivity was markedly highest in small airway epithelial cultures and lowest in bronchial SMG cultures (Figure 4C,D). These data support a hypothesis in which region-specific and disease-associated reductions in BPIFA1 expression constitute a key epithelial-intrinsic determinant of susceptibility to RSV and, potentially, other respiratory viruses.

### 3.4. BPIFA1-Derived Functional Peptides Protect Myhre Syndrome Airway Epithelial Cells Against RSV Infection

**Figure 5:**
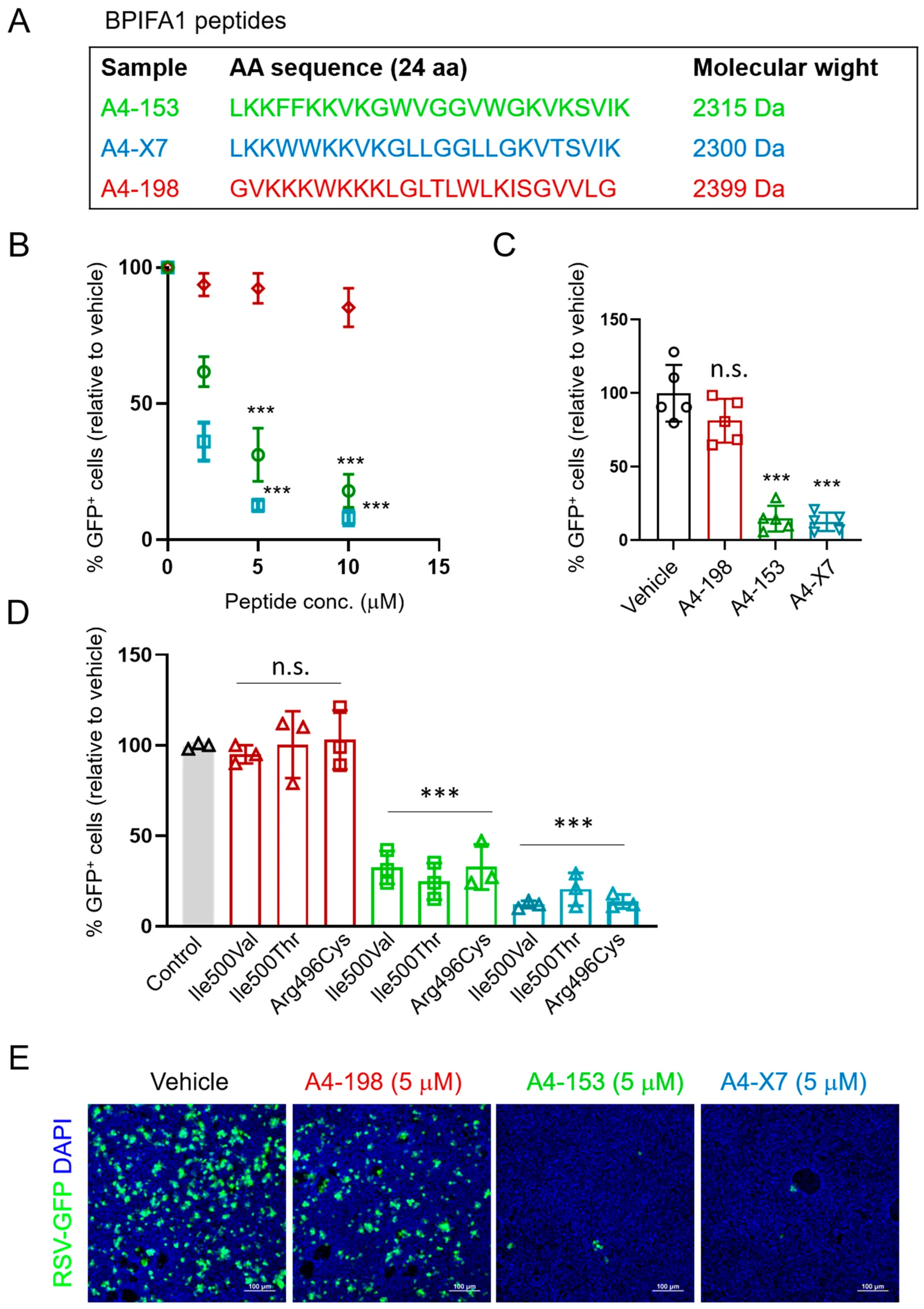
BPIFA1-derived functional peptides protect Myhre syndrome airway epithelial cells from RSV infection. (**A**) Amino acid sequences of the BPIFA1-derived peptides A4-153, A4-X7, and A4-198. (**B**) RSV was pre-incubated with each BPIFA1 peptide at indicated concentrations for 30 min before infection of healthy NEC ALI cultures. RSV-GFP-positive cells were quantified at 1 dpi and normalized to vehicle-treated controls. Data are presented as mean ± SEM; *** *p* < 0.001; *n* = 3 independent experiments. (**C**) RSV was pre-treated with each BPIFA1 peptide (5 μM) before infection of healthy NECs. RSV-GFP-positive cells were quantified at 1 dpi and normalized to vehicle-treated controls. Data are presented as mean ± SEM; *** *p* < 0.001; n.s.: not significant. *n* = 5 independent donors. (**D**) RSV was pre-treated with each BPIFA1 peptide (5 μM) before infection of Myhre syndrome NECs. RSV-GFP-positive cells were quantified at 1 dpi and normalized to vehicle-treated Ile500Val NECs (control). Data are presented as mean ± SEM; *** *p* < 0.001; n.s.: not significant. *n* = 3 independent experiments. (**E**) Representative immunofluorescence images of RSV-GFP-positive cells at 1 dpi showing that A4-153 and A4-X7 markedly reduced RSV infectivity in Ile500Val NECs, whereas A4-198 showed no significant reduction compared with vehicle-treated controls. Scale bars, 100 μm.

Full-length BPIFA1 (256 amino acids; ~25 kDa) is susceptible to proteolytic degradation following secretion into the ASL, and its size presents challenges for direct therapeutic application. To circumvent BPIFA1 deficiency in Myhre syndrome, we evaluated whether stabilized 24-amino acid BPIFA1-derived peptides could restore antiviral defense in NECs. We have previously characterized peptides A4-153 and A4-X7 as retaining full antimicrobial biological activity with enhanced resistance to enzymatic degradation relative to the native BPIFA1 sequence [37]. A scrambled control peptide, A4-198, was engineered to maintain similar charge and amino acid composition comparable to functional peptides while lacking biological activity (Figure 5A).

We first evaluated the antiviral activity of BPIFA1-derived peptides using one of the healthy NEC ALI cultures. Prior cytotoxicity studies demonstrated that peptide concentrations up to 10 μM did not produce detectable toxicity; therefore, concentrations within this range were used for all subsequent antiviral experiments. RSV-GFP (MOI = 1.0) was pre-incubated with individual peptides for 30 min before apical infection of NEC ALI cultures for 1 h. Following viral adsorption, the inoculum and peptide mixture were removed, the apical surface was washed, and GFP^+^ cells were quantified at 1 dpi as a measure of the ability of RSV to attach and infect the cells. The results demonstrated that A4-153 or A4-X7 peptide significantly reduced RSV infectivity in a concentration-dependent manner, as evidenced by a progressive decrease in the number of GFP^+^ cells. In contrast, the scrambled control peptide A4-198 exhibited no detectable antiviral activity (Figure 5B), indicating that the inhibitory effect is sequence-specific rather than a nonspecific peptide effect. Based on these encouraging findings, we next validated the antiviral activity using an expanded cohort of healthy donor-derived NEC ALI cultures (*n* = 5). The results confirmed that treatment with either A4-153 or A4-X7 at 5 μM significantly reduced RSV infection compared with vehicle and A4-198-treated controls, as reflected by a marked reduction in GFP^+^ cells at 1 dpi (Figure 5C). Next, we extended our studies to NEC ALI cultures derived from 3 patients with Myhre syndrome (Ile500Val, Ile500Thr, Arg496Cys). Consistent with the results obtained in healthy NECs, pre-incubation of RSV with either A4-153 or A4-X7 (5 μM) markedly reduced the number of GFP^+^ cells at 1 dpi (Figure 5D). In contrast, RSV pre-treated with A4-198 (5 μM) produced infection levels comparable to those observed in control (vehicle-treated Ile500Val NEC cultures) (Figure 5D). Figure 5E shows representative images of Ile500Val Myhre syndrome NEC ALI cultures at 1dpi, showing the robust antiviral activity of A4-153 or A4-X7. Together, these findings demonstrate that BPIFA1-derived peptides possess potent and sequence-specific antiviral activity against RSV and that this protective effect is preserved in BPIFA1-deficient Myhre syndrome airway epithelium.

## 4. Discussion

**Figure 6:**
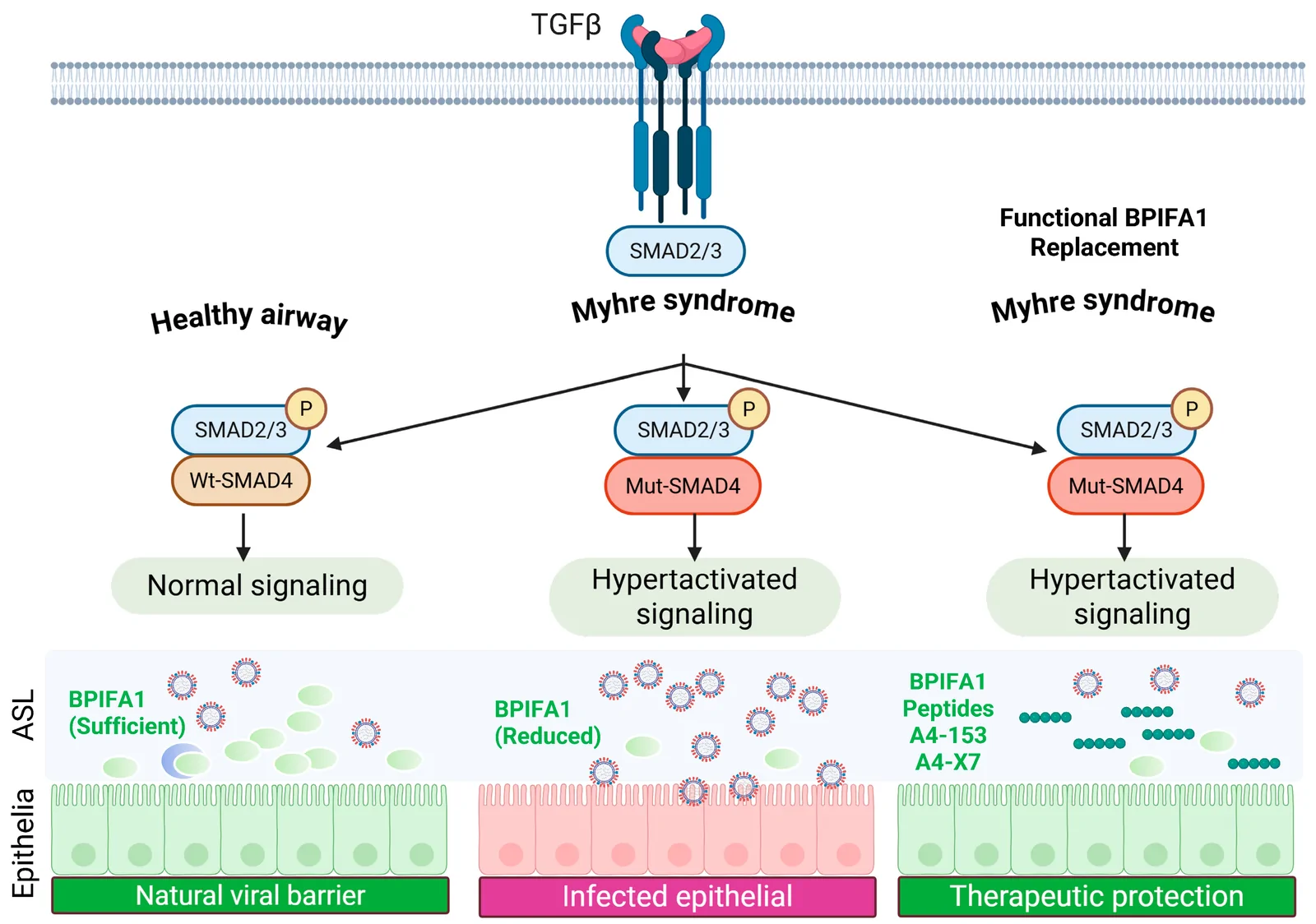
Proposed model of the SMAD4–BPIFA1 axis underlying respiratory viral susceptibility in Myhre syndrome and its therapeutic rescue. Under physiological conditions, controlled, regulated TGF-β/SMAD signaling maintains sufficient BPIFA1 expression in the airway surface liquid, thereby restricting RSV entry and spread while preserving epithelial barrier integrity. In patients with Myhre syndrome, gain-of-function SMAD4 mutations enhance TGF-β/SMAD signaling, suppress BPIFA1 expression, and promote increased RSV infectivity, viral dissemination, junctional disruption, and reduced barrier function. Stabilized BPIFA1-derived peptides (A4-153 and A4-X7) functionally compensate for endogenous BPIFA1 deficiency and restore antiviral protection. Whether SMAD4 suppresses BPIFA1 through direct transcriptional repression or indirect regulatory mechanisms remains to be determined.

In this study, we establish that GOF SMAD4 signaling drives a previously unrecognized epithelial-intrinsic mechanism of antiviral vulnerability in Myhre syndrome: SMAD4 gain-of-function → BPIFA1 suppression → increased RSV infectivity at an early stage (Figure 6). The results highlight the vital roles of pathological suppression of BPIFA1 expression in the airway epithelium with increased viral infections. Using patient-derived nasal ALI cultures, we demonstrate that Myhre syndrome NECs are markedly more susceptible to RSV infection and associated epithelial injury compared to healthy controls (Figure 6). We have shown that pharmacological activation of TGF-β1/SMAD signaling is sufficient to phenocopy this disease state in healthy donor cells, and that BPIFA1 deficiency induced either by SMAD activation or shRNA-mediated knockdown recapitulates the observed viral susceptibility phenotype. Critically, we demonstrate that stabilized BPIFA1-derived peptides can restore antiviral protection in Myhre syndrome NECs, providing proof of concept for a targeted therapeutic strategy (Figure 6).

Our findings extend prior observations that TGF-β signaling promotes viral pathogenesis across multiple respiratory infections [12,13,14,15,19,20,38,39]. The established pro-viral mechanisms of TGF-β—including suppression of interferon responses, cytoskeletal reorganization, and inhibition of cellular proliferation—collectively create a permissive environment for viral replication [12,13,14,15,19,20,38,39]. Our data now add BPIFA1 suppression as a distinct downstream effector of TGF-β pro-viral activity in airway epithelium. This finding is particularly significant in the context of Myhre syndrome, where constitutive amplification of SMAD4-mediated transcriptional activity chronically suppresses BPIFA1 in the ASL, the precise compartment where respiratory pathogens first encounter the host. BPIFA1 is a multifunctional innate defense protein enriched in the upper and proximal airways, where it exerts antimicrobial and immunomodulatory activities, including emerging antiviral functions. Our shRNA-mediated knockdown experiments demonstrate that loss of BPIFA1 alone is sufficient to markedly increase RSV infectivity and epithelial injury in healthy nasal epithelial cells, providing direct evidence that BPIFA1 is a crucial antiviral effector in the human airway.

Our data also provided a useful framework for understanding regional differences in viral susceptibility across the airway epithelium. The nasal and upper conducting airways, including the underlying submucosal glands, express high levels of BPIFA1, consistent with their critical roles in host defense against viral and bacterial infections. In contrast, small airway epithelial cells express little to no BPIFA1 and appear to possess more limited anti-infectious capacity, potentially reflecting a tradeoff that favors robust NRF2-dependent oxidative stress responses over frontline antimicrobial defense [40]. This spatial disparity also has important pathophysiological implications: when viral replication is not effectively contained in the upper airway and extends into distal lung regions, the relative absence of BPIFA1-mediated defense may permit enhanced viral propagation, leading to heightened inflammation, severe pneumonia, and downstream tissue injury.

BPIFA1 deficiency is not unique to Myhre syndrome. Reduced or dysregulated BPIFA1 expression has been reported in several chronic airway diseases, including cystic fibrosis, chronic obstructive pulmonary disease, and asthma [41], all of which are associated with increased susceptibility to respiratory viral infections and disease exacerbations. In addition, patients with Job syndrome (autosomal-dominant hyper-IgE syndrome caused by loss-of-function STAT3 mutations) frequently develop severe, life-threatening viral pneumonia [42,43], and we have demonstrated that primary airway epithelial cells from these patients exhibit markedly reduced BPIFA1 expression [44]. We have also previously reported that infant airway epithelial cells are intrinsically more susceptible to RSV infection than adult airway epithelial cells [35]. Whether this heightened susceptibility is associated with reduced BPIFA1 expression or impaired BPIFA1 secretion in infant airway epithelial cells remains unknown and warrants further investigation. Collectively, these findings suggest that BPIFA1 deficiency may represent a common epithelial vulnerability underlying increased respiratory viral susceptibility across diverse airway diseases and developmental stages. Accordingly, BPIFA1-derived peptide therapeutics aimed at restoring BPIFA1 function may have broad translational potential beyond Myhre syndrome.

The molecular mechanisms underlying BPIFA1-mediated antiviral protection against RSV remain incompletely defined and warrant further investigation. In IAV infection, BPIFA1 has been shown to directly interact with hemagglutinin (HA), competitively inhibiting virion attachment to sialylated receptors and thereby preventing viral entry [28]. In contrast, RSV infection does not rely on sialic acid-dependent attachment; instead, viral entry is mediated by interactions of the G protein with host receptors, such as heparan sulfate proteoglycans (HSPGs) and CX3CR1, and of the F protein with receptors such as nucleolin and IGF1R. Several non-mutually exclusive mechanisms may account for BPIFA1 antiviral activity in this setting. BPIFA1 may directly kill/neutralize RSV or bind RSV surface proteins (G and/or F), thereby blocking its ability to engage host cells. Alternatively, BPIFA1 may not impact RSV virions, but act primarily on the host cell surface, therefore shielding viral receptors from engagement. Supporting this model, our preliminary show that BPIFA1-derived peptides remain antiviral even after preincubation with epithelial cells and removal of unbound peptide, suggesting a potential receptor-shielding mechanism. Further comprehensive mechanistic studies will be required to delineate these pathways.

Several limitations of the current study merit consideration. First, our experiments utilized RSV as the primary viral model; whether the SMAD4–BPIFA1 axis similarly governs susceptibility to other clinically relevant respiratory viruses—such as human rhinovirus, influenza, SARS-CoV-2, or human metapneumovirus—remains to be determined and constitutes an important direction for future investigation. Second, the current studies were conducted in nasal ALI cultures, which, while highly physiologically relevant, do not fully recapitulate the microenvironment and immune cell interactions of airways. Future studies in ex vivo airway models containing both epithelial cells and immune cells, and ultimately in appropriate animal models, will be necessary to validate these findings in more complex biological contexts. Third, although we show that SMAD pathway activation is one of the key pathways in modulating BPIFA1 expression, the underlying regulatory mechanisms remain unclear. This effect may result from direct transcriptional repression via SMAD-responsive elements or indirectly through altered epithelial differentiation and loss of serous cell identity, the primary BPIFA1-expressing cell type. Fourth, due to the ultra-rare nature of Myhre syndrome, this study included NECs derived from only three patients carrying three distinct SMAD4 mutations. However, this limitation is mitigated by the consistent phenotype observed across all patient-derived cultures, as each exhibited a similar increase in viral susceptibility. Fifth, this study focused primarily on the very early stages of RSV infection. Future studies should evaluate the therapeutic efficacy of BPIFA1-derived peptides following viral attachment and entry to better define their clinical potential. Our preliminary data indicated that continued peptide treatment after removal of the RSV inoculum significantly reduced viral replication and cell-to-cell spread, suggesting that BPIFA1-derived peptides exhibit “therapeutic” antiviral activity along the course of viral infection.

Because prolonged incubation of peptide solutions disrupted the physiological ALI conditions and could induce epithelial stress and compromised barrier function. To better model therapeutic delivery, we are currently developing aerosol-based approaches to deliver BPIFA1-derived peptides while preserving the integrity of the ALI culture. Evaluation of these delivery strategies and the post-infection therapeutic efficacy of BPIFA1-derived peptides is the focus of future studies.

Our study demonstrates that GOF SMAD4 signaling impairs airway epithelial antiviral defense through suppression of BPIFA1, identifying the TGF-β/SMAD4–BPIFA1 axis as a key regulator of innate antiviral immunity in the human airway. These findings provide new mechanistic insight into the recurrent respiratory infections observed in Myhre syndrome and establish BPIFA1-derived peptides as a promising therapeutic strategy to restore antiviral protection. Although prevention options have expanded with three licensed vaccines, RSV treatment options remain limited [45], and a novel BPIFA1-derived innate defense peptide approach may provide a new therapeutic intervention. More broadly, the BPIFA1 insufficiency may represent a generalizable mechanism of viral vulnerability in airway diseases, warranting further investigation across a spectrum of chronic and genetic respiratory conditions.

## 5. Conclusions

This study identifies the SMAD4–BPIFA1 axis as a previously unrecognized, epithelial-intrinsic mechanism underlying viral susceptibility in Myhre syndrome. Using patient-derived airway epithelial cultures, we demonstrate that gain-of-function (GOF) SMAD4 signaling suppresses BPIFA1, thereby enhancing susceptibility to RSV infection. Loss of BPIFA1 alone recapitulates this hyper-susceptible phenotype, establishing BPIFA1 as a key downstream mediator linking aberrant SMAD4 signaling to impaired innate antiviral defense. Furthermore, an inverse correlation between BPIFA1 expression and RSV infectivity suggests that this mechanism extends beyond Myhre syndrome to other BPIFA1-deficient airway disorders. Crucially, treatment with stabilized, BPIFA1-derived peptides restored antiviral protection in both healthy and Myhre syndrome epithelium, providing proof of concept for peptide-based therapeutics. Together, these findings establish the TGF-β/SMAD4–BPIFA1 pathway as a driver of respiratory viral vulnerability and highlight BPIFA1 restoration as a promising strategy to combat RSV and related respiratory pathogens.

## Data Availability

The original contributions presented in this study are included in the article. The data presented in this study are available on request from the corresponding author.
